# Burnout Syndrome in forensic medicine and its association with vicarious trauma, posttraumatic stress syndrome and occupational stress

**DOI:** 10.1007/s00414-024-03398-7

**Published:** 2025-01-10

**Authors:** Lilioara-Alexandra Oprinca-Muja, Cosmin-Ioan Mohor, George-Călin Oprinca, Roxana Cardoș, Carmen-Daniela Domnariu, Adrian-Nicolae Cristian, Ciprian Băcilă, Sorin-Radu Fleacă, Alina Cristian, Silviu Morar

**Affiliations:** 1 Faculty of Medicine, Lucian Blaga University of Sibiu, Sibiu, 550169 Romania; 2 Department of Clinical Psychology, Babeș-Bolyai University of Cluj Napoca, Cluj- Napoca, 400347 Romania

**Keywords:** Burnout, Vicarious trauma, PTSD, Forensic medicine, Occupational stress, Maslach

## Abstract

The burnout phenomenon is a subject of considerable interest due to its impact on both employee well-being and scientific inquiry. Workplace factors, both intrinsic and extrinsic, play a pivotal role in its development, often leading to job dissatisfaction and heightened burnout risk. Chronic stress and burnout induce significant dysregulation in the autonomic nervous system and hormonal pathways, alongside structural brain changes. This paper presents a preliminary review of the literature on burnout syndrome among forensic science workers, focusing on the prevalence of this phenomenon and its triggers. This review aims to consolidate existing research on burnout among forensic medicine workers, highlight significant findings, and encourage further studies. Symptoms range from demoralization to somatic complaints. The Maslach Burnout Inventory (MBI) was the main tool in assessing burnout levels alongside measures of occupational stress, vicarious trauma and posttraumatic stress syndrome. Forensic medicine workers face unique stressors, with notable impacts on burnout levels. These workers experience challenges such as workplace conflicts and exposure to traumatic cases, leading to moderate or high burnout levels, particularly, emotional exhaustion, depersonalization or low personal accomplishment. Despite the prevalence of burnout, many forensic medicine workers lack access to support networks and perceive a disregard for their well-being from supervisors. There is a pressing need for further research to understand the biological mechanisms, susceptibility factors, and discover diagnostic markers of burnout syndrome, with the goal of its recognition as a psychiatric disorder in diagnostic manuals like the Diagnostic and Statistical Manual of Mental Disorders.

## Introduction and scope of this review

The phenomenon of burnout has garnered significant attention, both from employers grappling with their employees psychological distress, and from scientists seeking to understand its precise causes, the pathophysiological mechanisms, associated risk factors, any preventive measures, and variations in susceptibility among individuals [1, 2]. However, burnout syndrome represents an emerging psychological dysfunctionality that has surged in recent years, posing a significant threat not only to those affected but also to the normal functioning of contemporary society [3]. This is particularly evident in vital societal systems marked by high job-related stress, such as healthcare, law enforcement, the judiciary, and the military. For example, burnout scores in healthcare professionals range from 16 to 86%, with a mean of 57.4% [4]. Emergency room workers exhibit the highest prevalence, with a little over 50% of personnel affected [4]. These percentages often reflect high levels of variability depending on geographic area, expertise, and the specialty of the physician or nurse. In Kuwait, for example, burnout levels are among the highest, with 77% of workers experiencing high or moderate levels of burnout [5]. Additionally, stressful situations, such as the COVID-19 pandemic, significantly impact prevalence, with burnout peaking at 73% among healthcare professionals during this period [6]. The burnout syndrome within the context of forensic medicine has been studied at a small scale and it is insufficiently understood. Through this article, we aim to outline the research conducted so far within this field and draw the conclusions necessary to better understand this psychological phenomenon affecting many healthcare workers, including those from the departments of forensic medicine. We present a general overview of the specific field for this working population, as there are not enough studies to conduct a systematic review, but to adopt a more systematic approach, we formulated several questions to guide our literature review and generate more robust results:

- What is the prevalence of burnout in forensic medicine workers and what is the variation of emotional exhaustion (EE), depersonalisation (DP) and persional acomplishment (PA) between different work categories?
- What are the triggers of burnout in a forensic setting, and what are the coping mechanisms?
- What factors can positively influence burnout levels in forensic medicine workers (FMW)?
- Is there an association between burnout, vicarious trauma, and posttraumatic stress syndrome in FMW?
- What occupational stressors are representative for FMW?

## A theoretical background on burnout syndrome

### History and definition

In the 1960s, Freudenberger noted signs of psychological distress among workers in a detoxification clinic. By the 1970s, he published an article introducing the term “burnout” defining it as a state of exhaustion stemming from excessive demands on energy, strength, or resources [7]. Maslach and her colleagues developed the Maslach Burnout Inventory (MBI) during their study, establishing it as a prominent tool for diagnosing clinical burnout. They defined burnout as a syndrome characterized by emotional exhaustion and cynicism among individuals working closely with others. This condition manifests as emotional depletion and difficulty engaging at a psychological level, resulting in negative attitudes and feelings toward clients [8]. 

Burnout can also be understood as a psychological state of exhaustion stemming from prolonged exposure to work-related stressors without sufficient coping resources [9]. Initially recognized in the public sector, where human service employees could express emotional distress and relational issues, burnout has now been acknowledged to extend beyond the workplace. It affects students, homemakers, and even those who retire early, leading to physical and psychological fatigue. In recent decades, burnout has emerged as a global concern, prevalent across different countries and cultures. However, its classification as a medical or social issue varies [10]. Prior to Freudenberger’s research, air traffic controllers reported burnout due to factors like inadequate equipment, long shifts, and operational difficulties. A subsequent cohort study linked burnout in air traffic controllers to chronic exhaustion, hypertension, and psychiatric issues [11]. 

### The three dimensions of burnout

Based on research conducted by Maslach et al., burnout syndrome, as described by the workers interviewed, consists of three pillars: high emotional exhaustion, depersonalization or cynicism, and low personal accomplishment [8]. EE, a key aspect of burnout, signifies feeling drained after social interactions. DP, stemming from emotional exhaustion, involves negative attitudes or detachment from others. PA denotes reduced feelings of success at work. While debate exists about including this dimension in burnout diagnosis, the overall progression from emotional strain to exhaustion, depersonalization, and diminished self-accomplishment undermines motivation and work effort, reducing performance, productivity, and client satisfaction, while negatively impacting employee well-being [12]. 

### Causes

Burnout often arises from factors within the workplace. These factors can be categorized as intrinsic, relating to individual needs like autonomy and recognition, or extrinsic, involving aspects of the work environment. Inadequacies in these factors can lead to job dissatisfaction and increase the likelihood of burnout. Conversely, supportive environments and positive feedback from supervisors can mitigate the risk of burnout by enhancing job satisfaction [13]. Organizational factors play a more significant role in work-related stress and job dissatisfaction. Key organizational factors include workload, a sense of control over one’s job, recognition or rewards for work, the quality of interpersonal relationships among colleagues, and perceived fairness in the workplace. These factors collectively shape employees experiences and their susceptibility to burnout [14]. Healthcare workers, especially physicians, face intense pressure due to the demanding nature of their work and the high stakes involved in patient care. Factors such as high workload, long hours, administrative tasks, and inadequate resources, contribute to stress and burnout. Job dissatisfaction among physicians is linked to EE and DP [15]. Feelings of isolation or incompetence among colleagues worsen work-related stress. In some regions, low reward-to-demand ratios and resource inadequacies further contribute to burnout among healthcare workers [15]. In medical residents, emotional burden from caring for severely ill or dying patients adds to stress levels. Moreover, concerns about future career prospects, job security, quality of medical training, and conflicts with senior physicians contribute to their professional stress [16]. Unlike stress, burnout is gradual and insidious, characterized by exhaustion, lack of enthusiasm, cynicism, and reduced workplace efficacy [17]. Work-life balance challenges and sleep disorders further exacerbate burnout, particularly in high-demand professions like medicine where support is often insufficient [17]. Another significant stressor and generator of burnout for healthcare workers, especially physicians, is the liability and risk of patient litigation [18]. These stressors are frequently observed in the medico-legal environment. For example, psychiatrists working in closed settings such as hospital wards, prisons, or rehabilitation centers are at a greater risk of malpractice claims and complaints and are more prone to practice defensive medicine. The risk of liability is associated with anxiety, anger, and restlessness in this category of workers [18]. In forensic medicine workers, the risk of claims is also present, not due to patient care in a hospital setting but because forensic physicians bear immense judicial responsibilities. Diagnostic errors can impact police investigations, generate potential legal challenges, or expose individuals to media scrutiny. An incorrect cause of death can obscure a homicide or lead to the wrongful prosecution of an innocent person.

### Pathophysiological mechanism

The human body relies on systems like the autonomic nervous system (ANS) to respond to stress, activating the fight or flight response in seconds. Research suggests that individuals with burnout often show reduced vagal tone, indicating disturbances in this initial stress response system [19]. This disruption begins with acute stress, which suppresses the parasympathetic vagal system and elevates adrenaline/noradrenaline catecholamines. Prolonged stress exacerbates sympathetic activity while delaying parasympathetic recovery [20].

In individuals facing chronic stress and burnout, significant dysregulation occurs in the hypothalamic-pituitary-adrenal (HPA) axis, which activates within minutes in response to stressors. This dysregulation involves abnormalities in the secretion of adrenocorticotropic hormone (ACTH) and cortisol, resulting in either excessively high or low serum cortisol levels [21]. This variation can be attributed to constant hyperstimulation of the hypothalamus by the limbic system, initially resulting in high cortisol levels in the early stages of burnout. However, prolonged exposure leads to HPA axis inhibition through negative feedback, resulting in sustained low cortisol levels. Similar patterns are observed in prolactin levels, reflecting a bimodal distribution with individuals exhibiting either very high or very low levels [22]. Stressors activate components of the limbic system, such as the amygdala and hippocampus, prompting the hypothalamus to release high levels of corticotropin-releasing hormone (CRH). This action stimulates the pituitary gland to secrete ACTH, which in turn reaches the adrenal gland, leading to cortisol release. This cascade results in increased blood pressure, hyperglycemia, heightened energy, and immune system inhibition. Furthermore, stress prompts the adrenal cortex to release Dehydroepiandrosterone sulfate (DHEA-S), considered a counterbalancing hormone to cortisol. Individuals experiencing burnout typically display elevated serum DHEA-S levels compared to control groups [20]. In response to chronic stressors, dopamine release inhibition increases prolactin release from the pituitary gland, contributing to chronic fatigue syndrome. Additionally, serotonin, acting via 5HT1A receptors, links chronic stress to depression. The disproportionate response and disrupted homeostasis of the ANS and HPA axis can lead to psychosomatic symptoms such as chronic fatigue and cardiovascular changes, including blood pressure dysregulation and abnormal heart rates [22].

Heart rate variability (HRV), measuring the variability of intervals between heartbeats, is a well-studied biological parameter linked to burnout syndrome. Reduced HRV often predicts burnout symptoms and is indicative of decreased vagal tone [19]. Brain-derived neurotrophic factor (BDNF) is a pivotal component in the pathophysiology of burnout syndrome, crucial for brain tissue development, neuroplasticity, and memory consolidation while protecting against stress-induced neuronal damage. Recent research has highlighted a notable decrease in BDNF levels among individuals with burnout compared to control subjects [20, 23, 24]. In individuals experiencing burnout, elevated glucocorticoid levels from HPA axis dysregulation, coupled with reduced BDNF levels, lead to structural and functional changes in brain regions housing glucocorticoid receptors, primarily within the limbic system. These changes contribute to microstructural abnormalities, diminished neurogenesis, and heightened hypothalamic CRH secretion, establishing a positive feedback loop [20]. 

Advanced neuroimaging methods like PET-CT, MRI, fMRI, and EEG have unveiled structural and functional alterations in several brain regions among burnout sufferers. Primarily, these changes impact the limbic system structures such as the amygdala, the cingulate gyrus (anterior and posterior), the dorsolateral prefrontal cortex, the hippocampus, as well as the diencephalon and the basal ganglia [25]. Advanced sequencing methods and enhanced PCR analyses have illuminated the molecular underpinnings of burnout syndrome. They have uncovered disparities in genes like NR3C1 (glucocorticoid receptor gene), SLC6A4 (serotonin transporter gene), and BDNF (brain-derived neurotrophic factor gene). These variances, including diverse methylation patterns, directly influence the hypothalamic-pituitary axis [20]. NR3C1 gene encodes the glucocorticoid receptor (GR), which regulates the function of the HPA axis. Dysregulation of the HPA axis has been implicated in various conditions, including psychiatric disorders and chronic stress-related illnesses [26]. SLC6A4 encodes a membrane protein crucial for the transport of serotonin from the synaptic cleft to presynaptic neurons, playing a pivotal role in the regulation of the serotonergic system and influencing stress susceptibility. The 5-HTTLPR polymorphism in the promoter region of SLC6A4 has been linked to various psychiatric disorders, including general depression, as well as antenatal and postpartum depression [27]. BDNF gene encodes BDNF which was described earlier.

### Risk factors

When assessing risk factors for burnout syndrome, studies commonly categorize them into three main groups: socioeconomic, occupational, and psychological factors. Among these, occupational stressors, notably prominent in the medical field, significantly escalate the risk of burnout [28–31]. Among medical professionals, nurses experiencing high workloads, multiple employments, limited experience, or single status are at increased risk [30]. Specialty selection is also a crucial determinant, with surgical disciplines posing a higher risk compared to medical fields. Professionals in intensive care units and oncology face heightened vulnerability due to heavier workloads, elevated malpractice risk, and lower rates of favorable outcomes [29]. Social stressors, such as lacking psychological support from family or colleagues, also significantly impact emotional exhaustion and depersonalization [31]. Psychological symptoms like depression and anxiety play a critical role in the development of burnout [32]. Geographical patterns are evident in burnout rates among healthcare professionals, with higher levels observed in South and Eastern European hospitals compared to Scandinavian ones [29]. These geographical variations reflect differences in social development levels and the strength of public sectors, alongside financial stability and support offered to medical personnel in Scandinavian countries.

### Clinical aspects and means of diagnosis

Burnout syndrome is not classified as a psychiatric disorder in the DSM-V (2013). However, it is categorized as a “State of vital exhaustion” under “Problems related to life-management difficulty” in the ICD-10 by the World Health Organization [33]. Various scales have been developed to assess and diagnose clinical burnout, with the MBI being the most recognized and widely used. Other scales include the Burnout Measure (BM), Copenhagen Burnout Inventory (CBI), Oldenburg Burnout Inventory (OLBI), Shirom-Melamed Burnout Questionnaire (SMBQ), and its later version, the Shirom-Melamed Burnout Measure (SMBM). Sweden has introduced a disorder called “exhaustion disorder” in their version of the ICD-10 to facilitate a more accurate and rapid diagnosis of burnout [34]. Studies have identified psychophysiological symptoms characteristic of burnout syndrome compared to other psychiatric disorders such as major depressive disorder, anxiety disorder, or post-traumatic stress disorder (PTSD). These symptoms include demoralization, subjective incompetence, fatigue, increased irritability, loss of interest or pleasure, anxiety, derealization, changes in appetite, insomnia or hypersomnia, psychomotor agitation, feelings of worthlessness, diminished ability to think and memory impairment, recurrent thoughts of death, suicidal ideation or attempt, somatic complaints, and exaggerated startle response. While not exclusive to burnout, these symptoms are commonly observed in individuals experiencing this syndrome [35]. 

## Materials and methods

### Eligibility criteria

The inclusion criteria were: (1) research articles; (2) studies utilizing qualitative, quantitative, or mixed methods; (3) assessment of burnout alone or in conjunction with other work-related psychological issues; and (4) study cohorts consisting of forensic medicine workers, forensic science personnel working in medical examiners offices, and/or forensic healthcare workers.

The exclusion criteria included (1) articles not defined as research, such as reviews, short communications, and letters to the editor; (2) research articles not focusing on forensic medicine professionals; (3) research articles that deal also with other job related personnel beside forensic medicine professionals.

### Search strategy and study selection

**Fig. 1:**
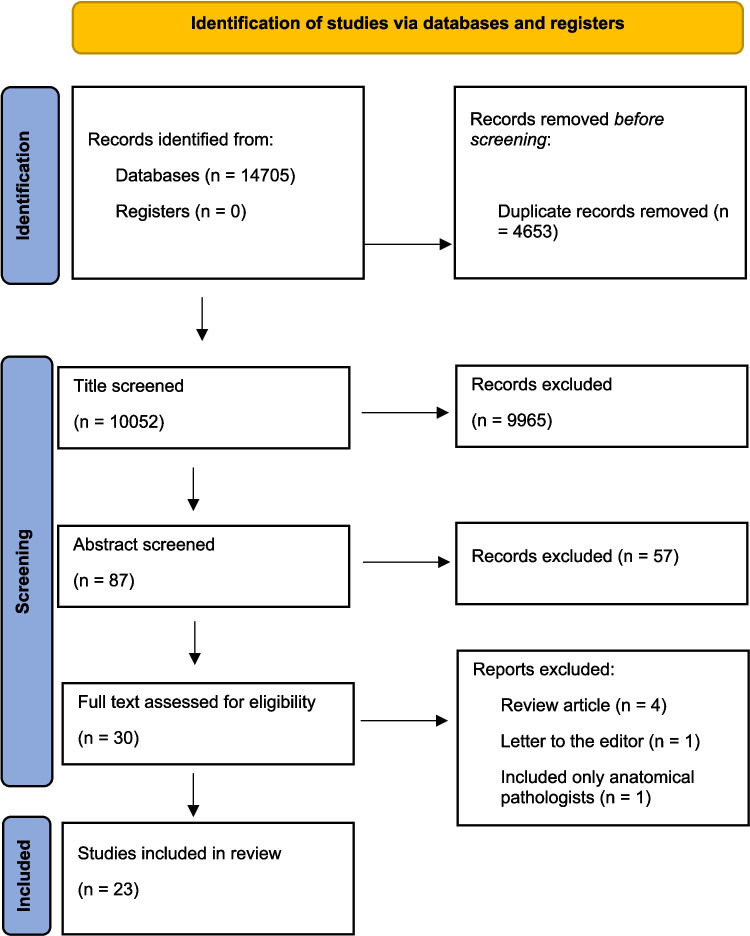
PRISMA flow

The study aimed to provide a comprehensive review of the articles on burnout among forensic medicine personnel. PubMed, Web of Science, ScienceDirect, Psychnet, and Google Scholar were used to compile a specialized literature database. After searching all databases, we retrieved 14,705 articles, from which 4,653 duplicates were removed. Two reviewers screened the titles of the remaining 10,052 articles, excluding 9,965 that did not pertain to forensic medicine personnel. Subsequently, another two reviewers screened the abstracts of the remaining 87 articles, removing 57 studies. The remaining 30 articles were assessed for eligibility, resulting in the exclusion of 7 studies: 4 were reviews, 1 was a letter to the editor, 1 included only anatomical pathologists, and 1 focused solely on law enforcement personnel (Fig. 1).

### Study cohorts characteristics

Our review encompassed diverse study groups within the field of forensic medicine, reflecting variations in departmental structures across different countries. These groups included forensic doctors (pathologists, medical examiners, coroners, sexual assault physicians, and forensic psychiatrists), forensic assistants, nurses, technicians (autopsy, mortuary, and laboratory staff), crime scene scientists, and faculty members. Seven interconnected groups emerged from the selected research articles: Forensic Science Personnel (FSP), Forensic Medicine Workers (FMW), and Forensic Healthcare Workers (FHW). The Forensic Science Personnel group included field and laboratory-based professionals such as death investigators, analysts, technicians, and faculty members. The Forensic Medicine Workers group focused on forensic doctors, forensic assistants, mortuary, and coronial staff. This group also included studies specifically addressing medical examiners (MEs) of sexual assault victims. The FHW group comprised mental health professionals like forensic nurses, psychologists, and psychiatrists, who often collaborate with other forensic medicine professionals.

### Articles characteristics

Our review identified eleven articles on burnout syndrome and twelve on occupational stress, with five covering both. Vicarious trauma (VT)and PTSD each had five articles, with two addressing both. Three articles explored burnout and coping mechanisms, while two focused on coping and vicarious trauma. Four articles discussed burnout and personal well-being, while four examined its relation to job satisfaction and two to alexithymia. Case exposure was a key focus in five studies, mostly regarding PTSD. Additionally, four articles explored the link between occupational stress or PTSD and mental/physical health disorders. Other aspects investigated included burnout and mindfulness, personality traits, and work support, each addressed in one article (Table 1).

**Table 1:**
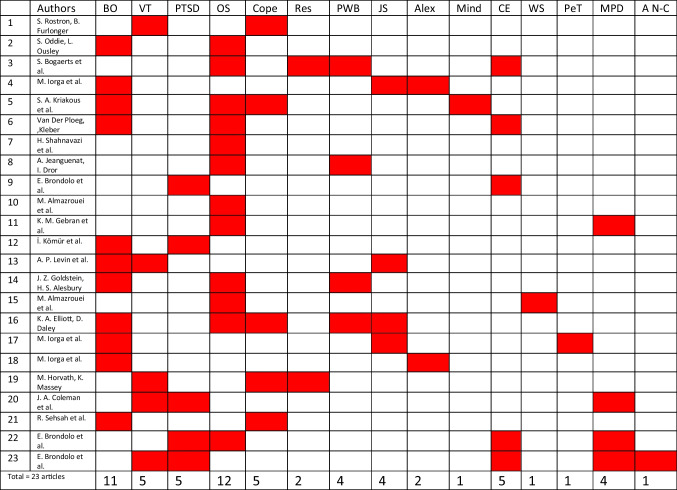
Psychological factors assessed by all articles reviewed

*BO *Burnout, *VT *Vicarious trauma, *PTSD *Posttraumatic stress syndrome, *OS *Occupational stress, *Cope *Coping, *Res *Resilience, *PWB *Personal well-being, *JS *Job satisfaction, *Alex *Alexithymia, *Mind *Mindfulness, *CE *Case exposure, *WS *Workplace support, *PeT *Personality traits, *MPD *Mental and physical disorders, *A N-C *Alienation, negative cognition

### Measurement tools from the reviewed articles

The reviewed studies employed diverse tools to measure burnout, including the MBI and the Professional Quality of Life (ProQOL) scale. Occupational stress was assessed using measures such as the Staff Stressor Questionnaire (SSQ), the Questionnaire on the Experience and Assessment of Work (QEAW), and the Psychiatric Nurse Occupational Stress Scale (PNOSS). VT was evaluated using instruments like the Trauma Attachment and Belief Scale (TABS) and the Trauma Symptoms Checklist-40 (TSC-40). PTSD was assessed using the Posttraumatic Stress Diagnostic Scale (PDS) and the Posttraumatic Symptom Screening Scale (PSSS). Alexithymia was measured using the Toronto Alexithymia Scale (TAS), while mental health disorders were evaluated with the Beck Depression Inventory-II (BDI-II) and the Beck Anxiety Inventory (BAI). Coping mechanisms were assessed using the Brief Cope Scale (BCI) and the Coping Strategies Indicator (CSI). Resilience was measured using the Resilience Evaluation Scale (RES) and the Connor-Davidson Resilience Scale (CD-RISK). Job satisfaction was evaluated using the Job Satisfaction Scale (JSS), and mindfulness was assessed using the Five Facet Mindfulness Questionnaire - Short Form (FFMQ-SF). Work support was explored through a personally developed questionnaire, while personality traits were analysed using the Big Five Inventory (BFI). Lastly, the Posttraumatic Cognitions Inventory (PTCI) provided insights into cognitive aspects related to trauma experiences (Table 2).

**Table 2:** Psychological factors assessed and questionnaires used by all articles reviewed

Questionnaire	BO	VT	PTSD	OS	Cope	Res	PWB	JS	Alex	Mind	CE	WS	PeT	MPD	A NC
TABS		1													
IES		2									2				
TSC-40		1													
CSI					1										
PNOSS				1											
MBI	9														
QEAW				2											
RES						1									
WBI							1								
C19FS				1											
TAS									2						
JSS								2							
SSQ				2											
BCI					4										
FFMQ-SF										1					
VT-ORG		1													
ProQOL	1	1						1							
CD-RISK						1									
PSSS			1												
3SQ							1	1							
BFI													1		
PB/GB-JWS		1													
BDI-II														3	
PDS			3												
BAI														1	
MOS-SF														1	
PSQI														1	
CES											1				
FCS		1													
PTCI															1

*BO *Burnout, *VT *Vicarious trauma, *PTSD *Posttraumatic stress syndrome, *OS *Occupational stress, *Cope *Coping, *Res *Resilience, *PWB *Personal well-being, *JS *Job satisfaction, *Alex *Alexithymia, *Mind *Mindfulness, *CE *Case exposure, *WS *Workplace support, *PeT *Personality traits, *MPD *Mental and physical disorders, *A N-C *Alienation, negative cognition, *TABS *Trauma and Attachment Belief Scale, *IES *Impact of Events Scale, *TSC-40 *Trauma Symptom Checklist – 40, *CSI *Coping Strategies Indicator, *PNOSS *Psychiatric Nurse Occupational Stress Scale, *MBI *Maslach Burnout Inventory, *QEAW *Questionnaire on the Experience and Assessment of Work; RES - Resilience Evaluation Scale; WBI - WHO-5 Well-Being Index; C19FS – Covid-19 fear scale; TAS – Toronto Alexithymia Scale; JSS – Job Satisfaction Scale; SSQ - Staff Stressor Questionnaire, *BCI *Brief Cope Inventory, *FFMQ-SF *Five Facet Mindful-ness Questionnaire - Short Form, *VT-ORG *Vicarious Trauma – Organizational Readiness Guide, *ProQOL *Profes-sional Quality of Life, *CD-RISK *Connor-Davidson Resilience Scale, *PSSS *Posttraumatic Symptom Screening Scale, *3SQ *Staff Support and Satisfaction Questionnaire, *BFI *Big Five Inventory, *PB/GB-JWS *Personal/General Belief in a Just World Scale, *BDI-II *Beck Depression Inventory II, *PDS *Posttraumatic Stress Diagnostic Scale, *BAI *Beck Anxiety Inventory, *MOS-SF *Medical Outcomes Study Questionnaire Short Form, *PSQI *Pittsburg Sleep Quality Inventory, *CES *Case Exposure Scale, *FCS *Family Contact Scale, *PTCI *Posttraumatic Cognitions Inventory

## Discussions

### Burnout in forensic medicine: prevalence, triggers, coping mechanisms, postive influencing factors and variations between different work categories

In a study by S. Oddie, L. Ousley, seventy one forensic healthcare workers were assessed for burnout using the PNOSS and MBI questionnaires. Findings revealed high levels of EE, moderate levels of DP, and low levels of personal PA. Occupational stressors such as limited resources and organizational issues had the greatest impact on EE, while organizational issues and staff conflict negatively influenced PA [36].

Two other studies explored burnout and occupational stressors in FHW, examining coping mechanisms, mindfulness, personal well-being, and job satisfaction. Kriakous et al. found that challenging patient behaviors, bureaucracy, lack of resources, low job status, and work conflict were associated with moderate levels of stress. High levels of mindfulness were linked to low-stress scores on the SSQ scale and low levels of burnout across all subscales. Prediction analysis illustrated that high levels of total occupational stress can generate high levels of DP but acting with awareness as a coping strategy can counteract DP. They also found that maladaptive coping strategies were predictive of high EE [37]. Elliott and Daley’s study involving 135 FHW, including those from forensic mental health institutions, revealed moderate levels of burnout across all three subscales of the MBI. Psychological distress, chronic stress, and negative coping strategies were associated with high levels of emotional exhaustion, which in turn reduces the overall psychological well-being of the individual. Also, higher levels of EE were observed in people consuming more alcohol. High levels of occupational stress and negative coping mechanisms predicted depersonalization, which was more prevalent in smokers. However, high levels of burnout in terms of PA were observed in only a fifth of the subjects, suggesting that the majority felt confident and rewarded in their work. Elevated occupational stress was associated with work-home conflicts, challenging patient behavior, low job status, lack of staff support, and bureaucracy [38]. 

Van Der Ploeg and Kleber investigated burnout syndrome and occupational stress among 132 forensic doctors using MBI, IES, QEEW, and CIS scales. They found that frequent acute stressors such as cases involving young children as victims of sexual assault, murder, suicide, aggressive detainees, or decomposed bodies were associated with a critical level of distress in 14% of subjects. There was a proportional relationship between the number of critical incidents and the level of posttraumatic response. Regarding burnout, 40.5% of subjects experienced high levels of DP, followed by EE at 25% and low PA at 20.2%. Individuals with high distress scores on the IES scale were at high risk for fatigue (33%) and burnout (44%). High emotional job demands, insufficient financial rewards, and lack of information from supervisors were identified as the most influential chronic stressors. Forensic doctors experienced more emotional distress, and received less job-related information, but were more satisfied with their financial rewards compared to a reference group. The study concluded that there is a cumulative effect of acute stressors in forensic doctors, with more exposure leading to increased symptoms of intrusion and avoidance [39]. 

J. Z. Goldstein and H. S. Alesbury conducted a study among FMW in a medical examiner’s office, revealing that 64% of staff felt worn out by work. Self-perceived burnout was associated with contact with human remains and next of kin of victims. Additionally, 50.7% of respondents lacked a current professional or academic community to discuss mental health concerns freely, and 73.9% felt that common mental health issues in their profession were not adequately addressed [40]. 

In the study involving mortuary staff, which included autopsy technicians, forensic medicine specialists, and residents, chronic stress was found to be triggered primarily by encountering pregnant women and infant victims in 64.1% of the subjects, followed by burned or fragmented corpses in 20.4% of subjects, and putrefied bodies in 15.5% of subjects. High levels of burnout were observed in terms of PA, with 76% of subjects experiencing burnout in this area. However, only 32% and 14% of subjects were diagnosed with high levels of burnout in EE and DP scales, respectively. There was no significant difference in burnout levels between male and female staff members, but autopsy technicians exhibited higher levels of EE, while forensic medicine residents showed lower levels of PA. When examining PTSD symptoms among mortuary staff, autopsy technicians were found to have higher scores for intrusive thoughts, physiological arousal, and avoidance compared to forensic medicine specialists or residents. This suggests that autopsy technicians may be at a higher risk of developing PTSD compared to other roles within the mortuary staff [41]. 

In the case of FSP, including both laboratory-based and field-based workers, burnout levels were moderate across all subgroups, with no significant differences observed between field-based and lab-based workers. Burnout was associated with low levels of organizational readiness concerning work-related exposures. The need to testify and work with victims families were identified as predictors of secondary traumatic stress among FSP [42]. 

The correlation between job satisfaction, alexithymia, and burnout was explored in three research articles by M. Iorga, all focusing on forensic pathologists and autopsy technicians [43–45]. In these studies, autopsy technicians exhibited lower levels of burnout across all three dimensions, with only 38.6% experiencing low PA. Additionally, only 4.5% of subjects showed definite signs of alexithymia, while 100% expressed high levels of job satisfaction. Critical job-related events and cases with high emotional impact were found to correlate strongly with alexithymia [43]. Exposure to cases involving children as victims of abuse, decomposing bodies, and inmate suicides contributed to emotional disturbance among forensic doctors. While forensic doctors generally exhibited low EE but high DP and low PA, those involved in teaching had higher levels of EE, but also higher PA. Higher job satisfaction was correlated with lower EE levels, while personality traits such as extraversion was associated with lower DP and neuroticism with higher EE [44, 45]. 

A comprehensive examination of burnout in relation to coping strategies was conducted by R. Sehsah et al. involving 133 forensic doctors, who completed the Brief COPE and MBI inventories. The findings revealed that 57.1% of forensic doctors experienced high levels of EE, 37.6% exhibited high levels of DP, and 66.2% reported low levels of PA. Moreover, 72.9% and 51.9% of participants demonstrated moderate to high levels of EE and DP, respectively, while 75.9% displayed moderate to low levels of PA. Factors associated with moderate to high levels of EE included stressful duties exceeding five per month, male gender, smoking habits, on-call obligations, and roles as forensic MEs. Similarly, individuals with moderate to high DP levels were predominantly young males (< 39 years), smokers, and forensic examiners with frequent stressful duties and on-call responsibilities. Notably, those with low PA were often female practitioners with less than 12 years of experience. Stressful duties reported included involvement in court proceedings, legal executions, exhumations, and cases of sexual assault and child victims. Forensic doctors with high EE tended to employ maladaptive coping mechanisms, while those with high DP levels often resorted to religion or behavioral disengagement. Conversely, individuals with low PA status were less inclined to use active coping strategies, exhibited lower levels of emotional focus, and engaged less in self distraction techniques [46]. 

Burnout prevalence among forensic medicine workers varies across its three dimensions. On average, 46.75% of individuals experience high levels of EE, 36.00% exhibit significant DP, and 50.23% report low PA. EE and DP levels tend to be higher in forensic doctors, autopsy technicians, and forensic science practitioners exposed to intense stressors.

Autopsy technicians exhibit higher levels of EE and intrusive thoughts, reflecting the intense psychological demands of their work. Forensic medicine residents, however, tend to report lower levels of PA, possibly due to the challenges of early career responsibilities. Field-based forensic science practitioners experience greater stress compared to their lab-based counterparts.

Stressors associated with burnout in FMW include challenging patient behavior, bureaucracy, exposure to traumatic cases such as child homicides and decomposed bodies, lack of resources, and inadequate workplace support. Coping strategies like mindfulness, social support, religion, and humor can help diminish these effects, while maladaptive strategies, such as substance abuse, are associated with poorer outcomes and heightened emotional distress.

Mindfulness has been shown to reduce EE, while acting with awareness helps decrease DP. High job satisfaction is associated with lower levels of EE, highlighting the protective role of workplace fulfillment. Additionally, resilience and personality traits like extraversion are linked to reduced DP, whereas neuroticism contributes to higher EE.

### Vicarious trauma, posttraumatic stress syndrome and burnout. Is there a pattern?

In the case of FMW, approximately 13% of workers were likely to have PTSD, 21% experienced mild to moderate depression, and approximately 10% had severe depression. Additionally, 23% experienced mild to moderate anxiety, with 7% experiencing severe anxiety. The latter was more prevalent in younger female individuals, while death investigators appeared more susceptible to depression and PTSD, and administrators to depression and anxiety compared to MEs [47]. Females were observed to be more susceptible to distress intolerance, depression, and PTSD [48]. E. Brandolo and his colleagues observed an association between case exposure in forensic personnel and symptoms of depression and PTSD [49]. Chronic exposure to traumatic cases seemed to raise levels of alienation and negative cognition, leading to PTSD and depression [49]. The most disturbing type of cases for FMW included infant or child homicide, or infant and child accidental death, significantly contributing to depression [47]. Frequent exposure to trauma and distressed family members triggered symptoms of alienation, distress intolerance, and negative cognition, crucial in the development of depression and PTSD [50]. Research on forensic examiners and nurses dealing with sexual assault found VT to be present on average with TAB-S scores ranging from extremely low to very high, with participants reporting higher levels of vicarious trauma in interviews compared to questionnaires. Problem solving, high social support, and avoidance strategies were the main coping mechanisms used in dealing with chronic stress. Also, the study demonstrated that older individuals tend to use more avoidance strategies and rely less on social support [51]. Horvath and Massey evaluated coping strategies and resilience to VT among 120 members of the Faculty of Forensic and Legal Medicine, finding humor, active coping, emotional support, positive reframing, self-distraction, behavioral disengagement, venting, religious behavior, but also substance abuse as frequent coping strategies [52]. Despite lower overall resilience compared to the general population, most respondents exhibited high levels of resilience, suggesting a preference for stressful and emotionally demanding professions. Lower beliefs in a just world and less resilience were associated with higher psychological distress [52]. 

Forensic staff exposed to tragic events often experience VT and PTSD symptoms, such as distress intolerance and avoidance. Psychological distress and cumulative exposure to traumatic events intensify the risk of burnout and PTSD, suggesting a cyclical relationship where unresolved trauma worsens burnout, which in turn exacerbates vulnerability to PTSD. Both VT and PTSD shares overlapping stressors with burnout such as case exposure to tragic events, emotional distress, and chronic workplace stressors.

### Occupational stressors in FMW

Apart from those previously outlined, occupational stress was referenced in six research articles from our database in relation to case exposure, medical or psychological conditions, as well as resilience, personal well-being, and workplace support. Two studies employed a personally designed questionnaire, while two others utilized interviews for assessing occupational stress. One article employed a theoretical narrative approach, while the final article utilized the QEEW and C19FS for measuring work-related stress, RES for resilience, and WBI for personal well-being. Results indicated that 36% of FSP experienced significant chronic stress primarily attributed to workplace scenarios, management/supervisors, or backlog pressure rather than personal life [53]. Notably, field-based workers exhibited higher stress levels than lab-based counterparts, with females reporting higher stress levels overall. Furthermore, 39% of workers believed that stress influenced their decision-making abilities [54]. In the case of FHW, S. Bogaerts et al. noted that individuals scoring low on WBI displayed heightened fear of COVID-19 during the pandemic, leading to increased psychosomatic symptoms and work-related stress. Also, individuals who had sleeping problems and who perceived their work more emotionally demanding reported a lower level of psychological well-being. Conversely, those with greater resilience reported higher psychological well-being, despite facing chronic workplace stressors such as insufficient personal resources, which often led to burnout [55]. During interviews with fourteen FMW, intense chronic workplace stress stemmed primarily from job structure, involving work scenarios related to death and various forms of violence. Workers also struggled to compartmentalize personal and professional lives, often becoming emotionally invested in cases. Inadequate building structures, secondary work overload, contamination risk from corpses, and the lack of protective equipment were identified as significant chronic stressors [56]. H. Shahnavazi’s findings from interviews with twelve forensic experts underscored that occupational stress, physical illness symptoms, depression, and psychological deterioration were predominantly linked to work and/or family conflict, interference with work duties, and the overall stressful environment within the Forensic Medicine department [57]. Additionally, employees felt their supervisors lacked concern for their physical and mental well-being, contributing to a detrimental cycle [57, 58]. 

## Conclusions

**Fig. 2:**
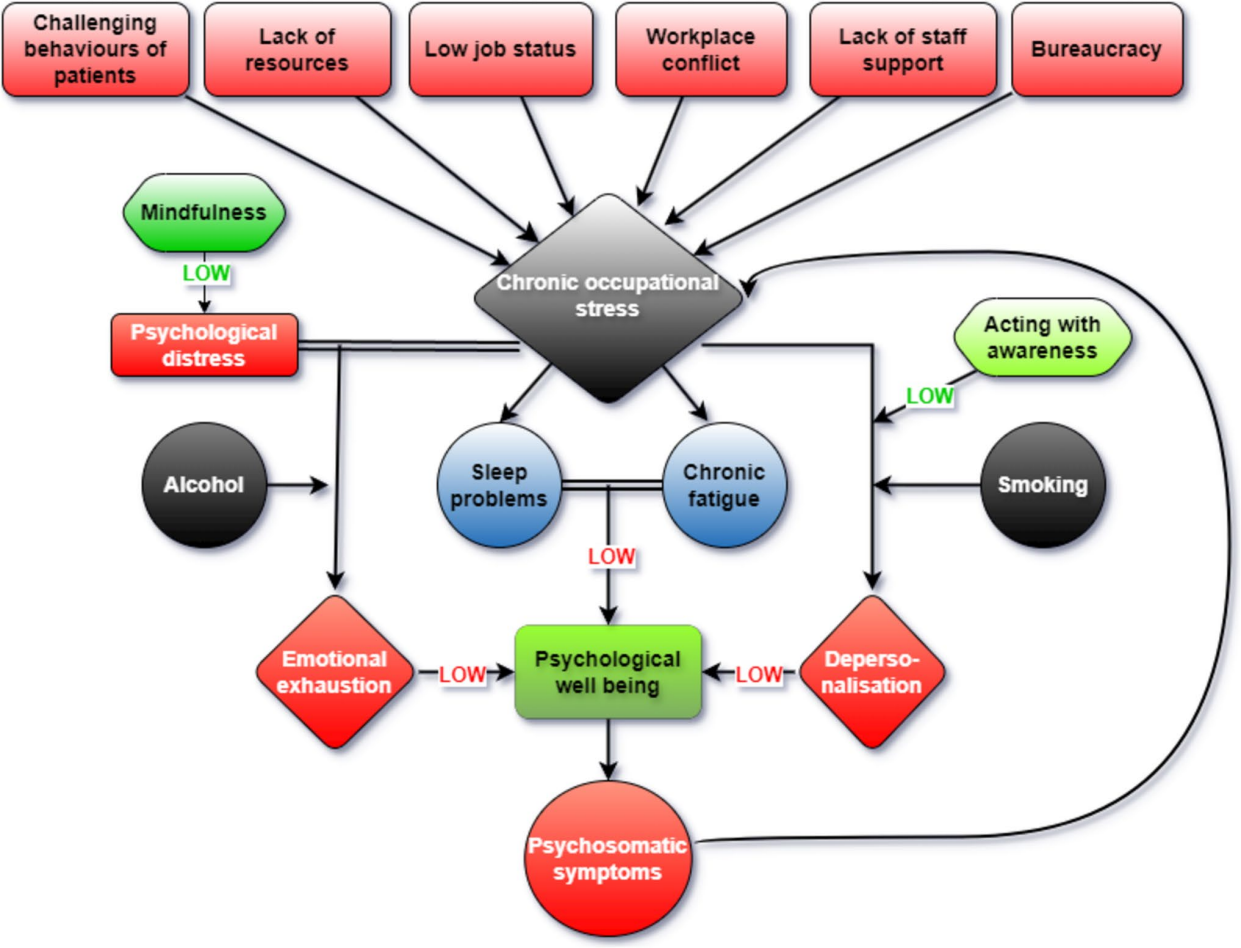
Evolution of burnout and occupational stress in forensic healthcare workers

The most prevalent occupational stressors in FHW working in psychiatric units include challenging patient behaviors, low job status, workplace conflicts, lack of appropriate resources, bureaucracy, and lack of staff support. Occupational stress in FHW can lead to sleeping problems and chronic fatigue, and those perceiving their work as more emotionally demanding may experience decreased psychological well-being, resulting in psychosomatic symptoms which in turn generates more chronic occupational stress. Greater resilience can mitigate the impact of low psychological well-being. FHW overall exhibits moderate levels of burnout across all three subscales, with a slight increase in EE. Chronic stressors such as organizational and administrative issues, along with high psychological distress and negative coping strategies like alcohol consumption, are associated with increased EE which in turn reduces psychological well-being. Negative coping strategies like smoking can also contribute to high levels of DP, while organizational issues and staff conflict negatively influence PA in FHW. High levels of mindfulness can substantially lower stress levels and mitigate burnout, while increased awareness can reduce DP levels in FHW (Fig. 2).

**Fig. 3:**
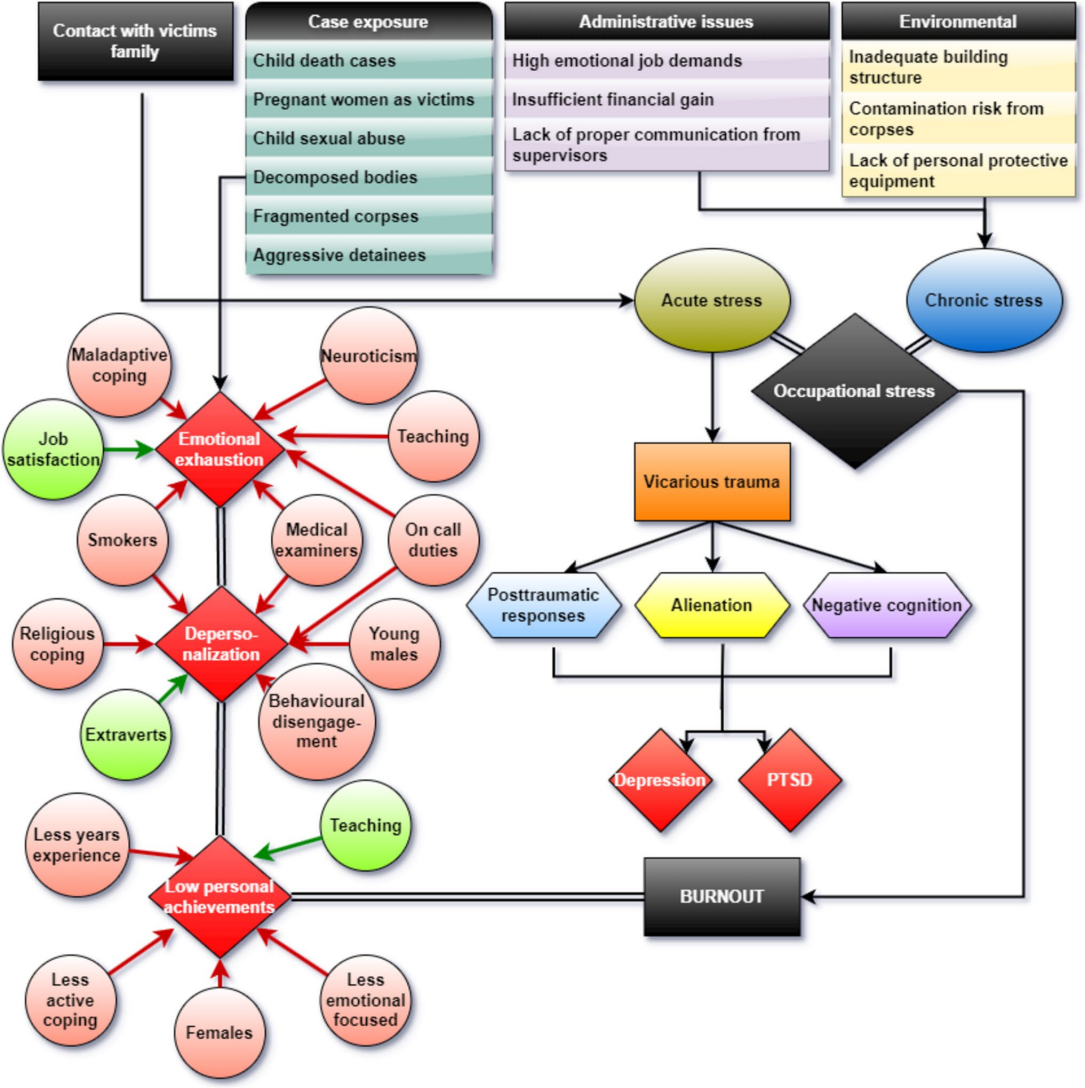
Factors influencing occupational stress, burnout, vicarious trauma and PTSD in FMW

FMW, including MEs, coroners, autopsy technicians, death investigators, and laboratory staff, face significant pressure due to the nature of their cases. The most prevalent acute occupational stressors include child death cases, pregnant women as victims, child sexual abuse cases, decomposed or fragmented corpses, and aggressive detainees. Chronic stressors often stem from administrative issues like high emotional job demands, inadequate financial compensation, poor communication from supervisors, inadequate building structures, contamination risks, and lack of personal protective equipment. Constant contact with victims families and frequent case exposure can lead to vicarious trauma, with approximately one out of six FMW experiencing critical levels of distress and posttraumatic responses, high levels of alienation, and negative cognition which in turn can generate PTSD or depression. Female workers, administrators, and death investigators are more prone to depression, anxiety, and PTSD. Autopsy technicians are particularly vulnerable to developing PTSD, experiencing frequent intrusive thoughts, and using avoidance techniques. Burnout is common among FMW, with nearly 50% experiencing high levels of DP, approximately one-fourth experiencing high EE, and around 20% experiencing low PA. Those dealing with frequent traumatic events are at higher risk of burnout, with approximately half experiencing high emotional distress. Overall, forensic doctors exhibit inconsistent burnout patterns, with some experiencing higher levels of DP and lower levels of EE, while others show the opposite pattern. EE levels tend to rise with increased case exposure, on-call duties, and maladaptive coping mechanisms, while high job satisfaction can mitigate EE. It is more frequent in smokers, in neurotic individuals, in FD who also teach, and in MEs. High DP levels are associated with frequent stressful cases, on-call duties and are more frequent in young male FD, smokers, forensic examiners, and those who use religion or behavioral disengagement as coping strategies. DP levels seem to be lower in extroverts. Low PA is more common in female forensic doctors with fewer years of experience and those employing less active coping strategies, are less emotionally focused, and use fewer self-distractions. FD who also teach have higher PA levels (Fig. 3).

In FMW dealing with sexual assault cases, VT levels vary widely, ranging from extremely low to very high. Coping mechanisms employed by these workers include humor, active coping, seeking emotional support, positive reframing, self-distraction, religious practices, venting, behavioral disengagement, and substance abuse. FMW of sexual assault cases exhibit high levels of resilience. Those with lower beliefs in a just world and lower resilience tend to experience higher psychological distress. We found no direct connection between VT or PTSD and burnout, acute stressors can generate VT and subsequently, PTSD and occupational stress related to both acute and chronic stressors can generate burnout (Fig. 3).

Better than half of FMW reported a lack of access to a professional or academic community where they could openly discuss mental health concerns associated with their job. Additionally, almost two-thirds of them felt that prevalent mental health issues within their profession were not sufficiently acknowledged. Moreover, FMW perceived a disregard for their physical and mental well-being by their supervisors, contributing to a detrimental cycle of neglect.

## Key clinical implications

The conclusions drawn from this review highlight the need for a multifaceted approach to managing and preventing burnout in forensic medicine workers, emphasizing the importance of both individual and organizational strategies. These strategies can comprise of:

- Early detection and intervention upon burnout by implementing regular screening for burnout symptoms in forensic medicine workers.
- Developing and providing access to comprehensive mental health support programs, including counseling, stress management workshops, and peer support groups which can help mitigate the impact of burnout.
- Addressing workload management by ensuring adequate staffing levels, reasonable work hours, and the distribution of case assignments.
- Offering ongoing professional development and training in stress management techniques, resilience building, and effective coping mechanisms.
- Promoting organizational changes that enhance job satisfaction, such as increasing job autonomy, providing opportunities for career advancement, and fostering a positive workplace culture, can contribute to lower burnout rates.
- Recognizing and addressing the potential for secondary traumatic stress and vicarious trauma in forensic medicine workers is essential. Providing training on how to manage exposure to traumatic material and offering support for those affected can reduce burnout risk.

## Future perspectives, research implications and limitations

During this comprehensive analysis of burnout syndrome, spanning from its historical roots and causative factors to its pathophysiological mechanisms, risk determinants, and evaluation among forensic medicine workers, we have gathered vital insights from specialized literature. It is critical to start research on burnout within forensic communities, investigating its correlation with various psychological factors such as vicarious trauma, PTSD, chronic occupational stress, depression, and anxiety, as well as physical illness. Despite the pivotal role of forensic medicine workers today, collaborating closely with the justice system to uphold a safe social environment, the phenomenon of burnout among them remains understudied. It is crucial to address the challenges confronted by these professionals and comprehend the ramifications of their demanding profession on their physical and psychological well-being. Furthermore, it is imperative to elucidate the precise pathophysiological mechanisms, triggers, biological predispositions, and brain anomalies, and to identify potential biomarkers for diagnosis. To this end, we propose large-scale research to encompass extensive study groups diagnosed with burnout, employing diverse assessment scales, and analyzing various blood biomarkers, in comparison with control cohorts. In addition, investigating the structural and functional aspects of different brain regions using state-of-the-art imaging techniques, coupled with molecular biology investigations to explore methylation patterns associated with burnout syndrome and genetic susceptibilities, is a necessity.

One limitation of this review is the exclusion of studies focusing on forensic medicine professions and jobs outside of the forensic field. While this approach was intended to minimize interpretation bias and ensure that the findings accurately reflect the unique experiences and challenges of forensic medicine professionals, it may have resulted in the loss of potentially relevant information. Future research should consider integrating findings from a wider range of occupational groups to better contextualize the stress and burnout dynamics in forensic medicine workers.

## Data Availability

All data generated or analysed during this study are included in this published article.
